# Implementation of a Cloud-Based Electronic Medical Record to Reduce Gaps in the HIV Treatment Continuum in Rural Kenya

**DOI:** 10.1371/journal.pone.0135361

**Published:** 2015-08-07

**Authors:** John Haskew, Gunnar Rø, Kenrick Turner, Davies Kimanga, Martin Sirengo, Shahnaaz Sharif

**Affiliations:** 1 Uamuzi Bora, Kakamega, Kenya; 2 University of Durham, Durham, United Kingdom; 3 British Antarctic Survey Medical Unit, Plymouth, United Kingdom; 4 Elizabeth Glazer Paediatric AIDS Foundation, Nairobi, Kenya; 5 National AIDS and STI Control Program, Nairobi, Kenya; 6 Ministry of Health, Nairobi, Kenya; Massachusetts General Hospital, UNITED STATES

## Abstract

**Background:**

Electronic medical record (EMR) systems are increasingly being adopted to support the delivery of health care in developing countries and their implementation can help to strengthen pathways of care and close gaps in the HIV treatment cascade by improving access to and use of data to inform clinical and public health decision-making.

**Methods:**

This study implemented a novel cloud-based electronic medical record system in an HIV outpatient setting in Western Kenya and evaluated its impact on reducing gaps in the HIV treatment continuum including missing data and patient eligibility for ART. The impact of the system was assessed using a two-sample test of proportions pre- and post-implementation of EMR-based data verification and clinical decision support.

**Results:**

Significant improvements in data quality and provision of clinical care were recorded through implementation of the EMR system, helping to ensure patients who are eligible for HIV treatment receive it early. A total of 2,169 and 764 patient records had missing data pre-implementation and post-implementation of EMR-based data verification and clinical decision support respectively. A total of 1,346 patients were eligible for ART, but not yet started on ART, pre-implementation compared to 270 patients pre-implementation.

**Conclusion:**

EMR-based data verification and clinical decision support can reduce gaps in HIV care, including missing data and eligibility for ART. A cloud-based model of EMR implementation removes the need for local clinic infrastructure and has the potential to enhance data sharing at different levels of health care to inform clinical and public health decision-making. A number of issues, including data management and patient confidentiality, must be considered but significant improvements in data quality and provision of clinical care are recorded through implementation of this EMR model.

## Introduction

Electronic Medical record (EMR) systems are increasingly being adopted to support the delivery of health care in resource-constrained settings and their implementation is particularly amenable to chronic diseases, including HIV. [1–4] The ultimate goal of an HIV program is to achieve sustained viral suppression and improved quality of life, for which an individual must be diagnosed as early as possible after infection, linked to care, remain engaged in care and receive antiretroviral therapy (ART) where medically indicated. [5]

Accessing HIV treatment is thus only part of the HIV care and treatment continuum, which is a long-term process that moves individuals through a set of stages beginning with HIV diagnosis and ultimately aiming to achieve durable viral suppression. However, at each stage, individuals may fall out of the HIV care and treatment continuum resulting in a “cascade” of reducing numbers of people living with HIV who remain healthy and well. [6] Some people do not join the continuum because they have not been diagnosed with HIV, those individuals who do test positive for HIV may not be effectively linked to care, [7] those linked to care may not receive ART once they are eligible, and many are not subsequently retained in care following diagnosis because of the absence of proactive interventions and support services. [6] These gaps undermine the public health impact of scaling up HIV treatment and reduce the proportion of people living with HIV with viral suppression. [8] Although the number of people receiving ART continues to rise in sub-Saharan Africa, it is estimated that around 75% of adults have not achieved viral suppression as a result of gaps at each stage described above. [5] In Kenya, an estimated 1.2 million people are living with HIV and national prevalence was 5.6% in 2012. [9] Only 40% of people living with HIV in Kenya had achieved viral suppression in 2012. [9]

Having built and expanded HIV treatment services, programme planners and implementers must now devote attention to closing these key gaps within the HIV care and treatment continuum. [6] Improved referral mechanisms and strengthened pathways of care are required for each stage of the treatment cascade to ensure effective linkage between testing and treatment, eligible patients receive ART, and to ensure patients are not lost to follow up when in care. [5,6,10] Few tools have been developed and implemented at scale, however, and effective linkage, initiation of ART, and retention in care remain a challenge. [11–14] In a systematic review, Rosen et al. did not find a single study that was able to follow a cohort of HIV-positive individuals from HIV testing to initiation of ART if they were not already eligible for ART when diagnosed. [7] Reasons for gaps within the HIV care and treatment continuum are multi-factorial but include systemic factors such as inadequate training and lack of access to timely and reliable data (electronic or paper based). [15–17] In most settings reviewed by Rosen et al., it was not possible to determine what happened to patients after testing positive for HIV, as there was no system in place to report whether further care and support had been received. [7]

Electronic medical record (EMR) systems for HIV can enable patients to be tracked and monitored throughout the HIV care and treatment continuum and need to be evaluated further in real-time program settings. EMR systems can help to strengthen pathways of care and close gaps in HIV care and treatment continuum by improving the quality of data collected to inform clinical and public health decision-making. [4,18–20] Improved access to and use of health information can then help to develop interventions to retain patients in care between testing and treatment and provide a deeper understanding of where and when individuals are being lost in the treatment cascade. Few studies have focused on EMR implementation in resource-constrained settings where unique challenges and barriers to implementation are encountered, [3,4] including limited human resources and the costs of equipment, software, and personnel. [21] Approaches to overcome these barriers are needed before EMR systems can support efficient, large-scale health care delivery in resource-constrained settings. [3]

Traditional models of EMR implementation have installed local systems infrastructure, such as a server and network in each clinic. Such locally installed infrastructure can be costly to implement and maintain and can be a barrier to EMR implementation in resource-constrained settings. [3] A local clinic model also limits the ability of the system to readily share information between different levels of health care. This underscores the need for innovative solutions that are appropriate for resource-constrained settings. In this study, we describe the implementation of a novel cloud-based EMR system for HIV care in Western Kenya and its impact on reducing gaps in the HIV care and treatment continuum.

## Methods

### Study setting

HIV outpatient care in Kenya is provided via a national system of Comprehensive Care Centres (CCC), through which counselling, treatment and other support services are provided for HIV. This study was conducted between October 2012 and November 2013 in the CCC of Kakamega Provincial General Hospital (PGH), Western Kenya. An electronic medical record system has been implemented in Kakamega PGH CCC since September 2009, which has the largest caseload of HIV patients in Kakamega County, Western Kenya.

### Electronic medical record

The EMR system–called Uamuzi Bora [22] (a Swahili phrase meaning “the right choice”)–was built using free, open source software and builds on common platforms and previous work, notably that of the Open Medical Record System (OpenMRS, https://www.openmrs.org). [23] The system was approved for use in Kenya by the Ministry of Health and National AIDS and STI Control Program (NASCOP) and adheres to national EMR standards. [24] The electronic patient record replicates information collected in the national paper-based HIV outpatient record, which includes socio-demographic information, patient source of testing, treatment history, and clinical and laboratory results.

The EMR system implemented a cloud-based model, rather than a local clinic model, which removed the need for local clinic infrastructure and enhanced data access and sharing at different levels of health care. In using the term “cloud-based” we refer to the fact that the server and data are hosted centrally and not by the individual clinic. The system used a secure virtual private network (VPN), provided by a mobile phone operator in Kenya, to which the server and clinic computers connected via a mobile data network. Google Chromebooks were used in the clinic, with built-in mobile data connection, to connect directly with the VPN. Clinic computers could not connect to the Internet (World Wide Web) and only those using SIM cards registered by the project could access the VPN. Daily copies of an anonymised version of the patient database were used to provide health information to different users at different levels of care via an online framework. Information provided by the online framework included demographics of new patient registrations (by day, age, gender and location), HIV status (by WHO stage, CD4 count) and by eligibility for ART. The server and data were hosted for the Ministry of Health at the Uamuzi Bora project office, located in Kakamega, to provide maintenance, security, and reliable power supply. The server connected to the VPN via a Worldwide Interoperability for Microwave Access (WiMAX) wireless communications standard and ran a customised version of OpenMRS on Ubuntu Linux.

### Data management and protection

The EMR system stored patient data securely and in accordance with best practices and all patients provided informed written consent prior to their data being entered into the system. If patients did not provide informed written consent their data was not entered in the system and they were not enrolled in the study. The server was physically secured in a locked office and access was limited to select project staff. Patient identifiable information was saved on an encrypted file system and the decryption key stored on removable media, which was held in a different secure physical location to the server. Connections between the clinic computers and the server used exclusively Hypertext Transfer Protocol Secure (HTTPS) over an Internet Protocol Security (IPsec) VPN. Project staff could also connect to the server from the internet using public-key authenticated Secure Shell (SSH), a cryptographic network protocol for secure data communication, over the IPsec VPN. All connection attempts to the server were logged and audited.

Encrypted backups were made of patient identifiable data and held for six months for the purpose of disaster recovery, after which time they were securely deleted. Daily anonymised versions of the database were created automatically by the server, which contained no patient identifiable data. This anonymous database was encrypted and transferred over the VPN to a public webserver, exposing an application programming interface (API), which allowed partners to access aggregated data. [25] Only prior anonymised patient data was used in the analysis for this study.

### Implementation and data collection

Data collection occurred over two phases. The pre-intervention phase took place between September 2009 and October 2012, when existing paper-based outpatient records were entered into the EMR system but no EMR-based data verification or clinical decision support was implemented. During this phase, data were double entered from the paper record into the EMR system using data-entry assistants recruited for the study.

During the intervention phase, between October 2012 and November 2013, EMR-based data verification and clinical decision support features were activated. Any new patients continued to be entered into the EMR system during this time and every patient visiting the CCC was eligible for inclusion into the EMR. All new patients consented to their data being entered into the EMR system during the study period.

### Data verification and clinical decision support

Electronic data verification and clinical decision support reminders were provided both during the consultation (to the clinician) and after the consultation (to the EMR study co-ordinator and data team). Automated structured query language (SQL) queries were programmed within the EMR (using the Flags module of OpenMRS) that enabled data verification and clinical decision support reminders to be displayed to clinicians during the consultation on the Google Chromebook. This reminder would remain on the patient record until corrective action was taken (such as missing data entered, patient initiated ART or patient returned to clinic). These SQL queries identified records that contained missing data, patients eligible for ART but not on ART (based on national guidelines at the time of CD4 count ≤350 cells/μl or WHO clinical stage 3 or 4 [26]) and patients who had missed appointments (they had missed their next appointment date by more than two weeks and no follow-up had been initiated by the clinic). Clinical staff were provided training in how to interpret and act on the data verification and clinical decision support flags and reminders appropriately. Automated SQL queries of all patient data were also run each day on the server and daily lists of patient IDs for each reminder were produced to highlight the results of these queries. The patient ID would remain in each daily list until corrective action was taken to remove the flag. The EMR study co-ordinator and two data assistants reviewed these reports with clinical staff to correct missing data, review eligibility for ART and contact patients who had missed clinic appointments.

### Costing information

The unit cost for the server and each clinic deployment, as well as subsequent maintenance and support costs, were calculated from overall budget expenditure over the duration of the project.

### Statistical analysis

Outcomes for the study were agreed prior to the start of the study. These included completeness of the patient record according to selected demographic and clinical variables, patients eligible for ART who had not yet started ART and contact of patients who had missed clinic appointments. Selected demographic (gender, date of birth, patient source) and clinical variables (first CD4 count and first WHO clinical stage) were chosen for review during data verification. Patient eligibility for ART was determined according to national guidelines, which states all HIV patients with a CD4 count ≤350 cells/μl or WHO clinical stage 3 or 4 should be initiated on ART. [26] Patients were classified as having missed a clinic appointment if they had missed their next appointment date by more than two weeks and no follow-up had been initiated by the clinic.

Demographic and clinical data of patients were compared using frequencies. The impact of the EMR system on missing data, eligibility for ART and missed clinic appointment was assessed using a two-sample test of proportions. All analyses were carried out using STATA version 12.1 (Stata Corp; College Station, TX).

### Ethics statement

Patient consent for participation was obtained by the clinical officer or nurse at each clinic consultation. The study was approved by the institutional review board at Kakamega Provincial General Hospital and the Director of Public Health, Ministry of Health, Kenya.

## Results

A total of 4,544 active HIV patients were registered in the EMR system at Kakamega PGH CCC before the start of implementation of data verification and clinical decision support in October 2012 (Table 1). Around one third of these patients were aged between 35 to 44 years and two-thirds were female. Around half of active patients tested positive for HIV at voluntary counselling and testing (VCT) services. Nearly half of active patients had a first CD4 count less than 350 cells/μl on registration and less than 20% of active patients did not have first CD4 count recorded in the patient record. More than 10% of active patients were registered with first WHO clinical stage 3 or 4 and nearly half of active patients did not have first WHO clinical stage recorded in the patient record. 2,127 patients were on ART and 1,346 of patients were eligible for ART, based on CD4 count ≤350 cells/μl or WHO clinical stage 3 or 4, but had not yet started ART (Table 1).

**Table 1:** Demographic and clinical variables of active patients registered in the EMR system pre- and post-intervention.

	Pre-intervention N. (%)	Post-intervention N. (%)
**Total**	4,544 (100.0%)	4,344 (100.0%)
**Age Group**		
<15years	412 (9.1%)	387 (8.9%)
15-24years	188 (4.1%)	223 (5.1%)
25-34years	1,011 (22.3%)	918 (21.1%)
35-44years	1,464 (32.2%)	1,402 (32.3%)
45-54years	930 (20.5%)	936 (21.6%)
55-64years	406 (8.9%)	399 (9.2%)
>65years	91 (2.0%)	78 (1.8%)
Missing	42 (0.9%)	1 (0.02%)
**Gender**		
Male	1,606 (35.3%)	1,489 (34.3%)
Female	2,894 (63.7%)	2,854 (65.7%)
Missing	44 (1.0%)	1 (0.02%)
**Patient source of testing**		
VCT	2,313 (50.9%)	2,241 (51.6%)
Outpatient	197 (4.3%)	301 (6.9%)
MCH-ChildClinic	5 (0.1%)	14 (0.3%)
PMTCT	225 (5.0%)	281 (6.5%)
Inpatient	295 (6.5%)	249 (5.7%)
TBclinic	113 (2.5%)	111 (2.6%)
Unknown	19 (0.4%)	187 (4.3%)
Other	1,002 (22.1%)	891 (20.5%)
Missing	375 (8.3%)	69 (1.6%)
**First CD4 count**		
≤350cells/μl	2,219 (48.8%)	2,435 (56.1%)
>350cells/μl	1,499 (33.0%)	1,555 (35.8%)
missing	826 (18.2%)	354 (8.2%)
**First WHO clinical stage**		
WHOStage1	707 (15.6%)	1,202 (27.7%)
WHOStage2	955 (21.0%)	1,601 (36.9%)
WHOStage3	585 (12.9%)	977 (22.5%)
WHOStage4	39 (0.9%)	85 (2.0%)
Missing	2,258 (49.7%)	479 (11.0%)
**ART status**		
EligibleandonART	2,127 (46.8%)	3,129 (72.0%)
EligiblebutnotonART*	1,346 (29.6%)	270 (6.2%)
NoteligibleandnotonARTorMissing	1,071 (23.6%)	945 (21.8%)

^*^ patients eligible for ART, based on CD4 or WHO stage, but who have not started ART

A total of 4,344 active HIV patients were registered in the EMR system at Kakamega PGH CCC at the end of the intervention period in December 2013 (Table 1). Around one third of these patients were aged between 35 to 44 years and two-thirds were female. Just over one half of active patients tested positive for HIV at voluntary counselling and testing (VCT) services. More than half of all active patients had a first CD4 count less than 350 cells/μl on registration and less than 10% of active patients did not have first CD4 count recorded in the patient record. One quarter of active patients were registered with first WHO clinical stage 3 or 4 and around 10% of active patients did not have first WHO clinical stage recorded in the patient record. 3,129 patients were on ART and 270 of patients were eligible for ART, based on CD4 count ≤350 cells/μl or WHO clinical stage 3 or 4, but had not yet started ART (Table 1).

The impact of EMR-based data verification on selected missing data pre- and post-intervention was assessed using a two-sample test of proportions (Table 2). A total of 2,619 patients were eligible to receive data verification reminders at the start of the implementation period and all 2,619 reminders were displayed constantly in the electronic patient record (to the clinician), or generated as a patient list each day (for the data team), until the missing data were corrected. An overall 40% (95% CI 38.2–41.8, p < 0.001) difference in missing data (age, gender, patient source of testing, first CD4 count or first WHO clinical stage) was recorded pre- and post- implementation of EMR-based data verification (Table 2). Significant difference in the amount of missing data for patient source of testing (6.6%, 95% CI 5.7–7.5), first CD4 count (10.0%, 95% CI 8.6–11.4) and first WHO stage (38.7%, 95% CI 36.9–40.4) were recorded pre- and post- implementation of EMR-based data verification (Table 2).

**Table 2:** Missing demographic and clinical variables of active patients registered in the EMR system pre- and post-intervention.

Missing Data	Pre N. (%)	Post N. (%)	% diff ^*^	95% CI	P
Age	42 (0.9)	1 (0.02)	-0.9%	0.6–1.2	<0.001
Gender	44 (1.0)	1 (0.02)	-0.9%	0.6–1.2	<0.001
Patient Source	375 (8.3)	69 (1.6)	-6.6%	5.7–7.5	<0.001
First CD4	826 (18.2)	354 (8.2)	-10.0%	8.6–11.4	<0.001
First WHO clinical stage	2,258 (49.7)	479 (11.0)	-38.7%	36.9–40.4	<0.001
Total ^**^	2,619 (57.6)	764 (17.6)	-40.0%	38.2–41.8	<0.001

^*^ Two-sample test of proportions

^**^ Patient records missing either age, gender, patient source, first CD4 or first WHO clinical stage data variables

The impact of EMR-based clinical decision support on numbers of patients eligible for ART, but not yet started on ART, was assessed by comparing frequencies pre- and post-intervention using a two-sample test of proportions (Table 3). A total of 1,346 patients were eligible but not on ART at the start of the implementation period and all 1,346 reminders were displayed constantly in the electronic patient record (to the clinician), or generated as a patient list each day (for the data team), until a clinical decision regarding treatment was taken. Almost one third of patients prior to introduction of EMR-based clinical decision support were eligible for ART, based on CD4 count ≤350 cells/μl or WHO clinical stage 3 or 4, but not yet started on ART. Post-implementation of EMR-based clinical decision support, 6% of patients were eligible but not on ART, representing a significant 23% (95% CI 21.9–24.9, p < 0.001) difference in patients eligible but not on ART (Table 3).

**Table 3:** Patients eligible but not on ART and patients followed up following a missed clinic appointment pre- and post-intervention.

Variable	Pre N. (%)	Post N. (%)	% diff ^*^	95% CI	P
Eligible but not on ART ^**^	1,346 (29.6%)	270 (6.2%)	-23.4%	21.9–24.9	<0.001
Patient follow-up ^***^	1 (0.02%)	656 (15.1%)	+15.1%	14.0–16.1	<0.001

^*^ Two-sample test of proportions

^**^ patients eligible for ART, based on CD4 count ≤350 cells/μl or WHO clinical stage 3 or 4, but who have not yet started ART

^***^ patients who have missed next appointment date by more than two weeks (as % of patients registered)

The initial, one-off unit cost for each outpatient clinic was approximately USD$ 500, including the cost of the Google Chromebook, clinic set-up and training costs. Subsequent maintenance and monitoring costs amounted to around USD$ 50 per clinic per month. The initial unit cost for the central server, online framework and connection was approximately USD$ 6,000 and subsequent maintenance costs were around USD$ 200 per month. This cost remained the same regardless of the number of additional units (outpatient clinics) that are included in the network. In addition to these system costs, one EMR supervisor (cost of USD$ 500 per month) was recruited to monitor clinic activities and provide support to clinicians and one IT systems administrator (USD$ 500 per month) was recruited to oversee the server and network.

## Discussion

The HIV care and treatment continuum must provide services that promote health and ensure individuals are retained across the treatment cascade in order to achieve durable viral suppression. [6] In this study, we describe the implementation of a novel cloud-based EMR system for HIV and its impact on reducing gaps in the HIV care and treatment continuum. Significant improvements in data quality and provision of clinical care patients are recorded through implementation of the EMR system, helping to ensure patients who are eligible for HIV treatment receive it early.

The cloud-based model of EMR implementation has the potential to provide real-time access to anonymised data beyond the level of the clinic, to inform timely HIV program decision-making. A program of follow-up was initiated using the EMR system, during which clinic-based peer counsellors reviewed patient records who had missed a clinic appointment and contacted them by telephone, but follow-up comparisons cannot be made. A total of 656 active patients were contacted through this program, of whom 323 patients agreed to return to clinic. This program of follow-up provides one example by which access to real-time data can be used to develop informed evidence-based programs. Further studies are encouraged to explore the potential applications of EMR system implementation for HIV programming in real-time program settings.

A cloud-based model of implementation may be able to remove many barriers to EMR adoption in resource-constrained settings, including the need for local clinic infrastructure, and has the potential to enhance data sharing at different levels of health care to inform clinical and public health decision-making. The cloud-based model may be more cost-effective and scalable than local clinic models as only one server needs to be maintained, secured and operated, without the cost of establishing local infrastructure including a server, network, security, maintenance and power supply in each clinic. The cloud-based model may also be able to offer economies of scale as the server and VPN network can function across wide geographic areas and a large number of clinics. Such an implementation model could be particularly suitable for remote and low volume sites and could enable and sustain large-scale implementation in resource-constrained settings.

A limitation of this implementation model in other settings is the need to take into account the mobile data network infrastructure as well as local guidelines on data management and security. Other limitations of the study include the short study period and not controlling for other potential confounding factors that may have influenced the effect of cloud-based EMR data validation and clinical decision support pre and post-implementation, such as improvement in training or clinical practice during the study period. Further work is encouraged to compare the cloud-based EMR model with local clinic-based EMR systems, including impact on cost-effectiveness, data completeness and data sharing and to consider the scalability of cloud-based models in settings where data network infrastructure is functional.
